# Crowding Out Effects of Alcohol Consumption Expenditure on Household Resource Allocation in Malawi

**DOI:** 10.1371/journal.pone.0263330

**Published:** 2022-02-04

**Authors:** Aubrey Jolex, Ben Kaluwa

**Affiliations:** Department of Economics, Chancellor College, University of Malawi, Zomba, Malawi; Universidad Adolfo Ibanez, CHILE

## Abstract

The study used Quasi maximum likelihood estimation (QMLE) on a nationally representative household level data set to estimate the effect of alcohol consumption expenditure on a set of expenditure proportions of other commodities. The results indicate that, the low-income, including the rural population, spent proportionately more on alcohol than their well-off and urban counterparts. Furthermore, the consumption of alcohol crowded-out expenditures on consumer non-durable (food and beverages), durable (housing) and essential services (education). The crowding out of these expenditures clearly has negative impacts on the wellbeing of individuals within households and communities through misallocated household resources. The strong, unequivocal message coming out of the results obtained in this study is that certainly for poorer countries alcohol consumption is inimical to household poverty reduction.

## 1 Introduction

Alcohol presents two sided effects. On one side, alcohol production and consumption can play an important role in generating personal and national incomes through employment. On the other side alcohol consumption exerts a burden across the health, social and economic systems whose costs can far outweigh the income benefits [1].

Alcohol consumption causes morbidity and mortality on a level with measles and malaria and at a higher rate than tobacco [2]. For example, non-communicable diseases in many low and middle-income countries such as South Africa, Mozambique, Zimbabwe, and Botswana are largely a consequence of the consumption of alcohol [3, 4]. Alcohol consumption kills more than 3 million people each year worldwide [5]. Given that most developing regions are characterized by hazardous drinking patterns, this results in heavier future burdens as the countries lack financial resources to provide adequate health and nutrition for their population [6].

In addition to the health risks associated with alcohol consumption, there can also be serious social and economic consequences for the consumer and the society at large [7, 8]. Alcohol is an addictive good and can potentially distort household budget.

Alcohol consumption and its correlates, poverty and poor nutrition suggest that expenditure on alcohol can constitute a significant part of household budget, reducing spending on basic needs such as food, healthcare, education, housing, transport, and energy among others [9–12]. This is referred to as the crowding-out effect, which can worsen poverty level and general well-being of households [13–16]. If this negatively affects investments in children’s nutritional status and education it results in what may be termed “intergenerational bargain failure” and the future wealth of poorer nations [17, 18].

Alcohol is the most widely consumed drug in the world. About half of the population above 15 years world-wide had consumed alcohol in the year of 2012 [19]. Total alcohol per capita consumption in the world’s population over 15 years of age rose from 5.5 litres of pure alcohol in 2005 to 6.4 litres in 2010, and was still at the level of 6.4 litres in 2016 [20].

Statistics also show increasing alcohol consumption in the population in Africa [21, 22]. Although some developing regions in Africa still practice abstention, many are characterized by hazardous drinking patterns. The region has the highest prevalence of heavy episodic drinking of at least 60 grams of pure alcohol on at least one occasion in the past 30 days. The region had 46% of women and 59% of male drinkers engaging in weekly heavy episodic drinking [23].

In the southern African context Malawi’s alcohol consumption levels are said to be similar to neighboring countries such as Zambia, and South Africa but slightly higher than countries like Kenya, Zimbabwe and Namibia [24].

In Malawi alcohol is consumed by both males and females mainly from the age of 15 and above [20]. According to a 2009 nationwide STEPS survey among adults (aged 25 to 64) in a sample of 5206 participants 30.1 percent of males and 4.1 percent of females consumed alcohol while 19.2 percent and 2.3 percent of males and females respectively were considered heavy drinkers [25].

Allocation of household resources to alcoholic drinks in Malawi is substantial; according to the Fourth Integrated Household Survey (IHS4), 6.8% of the sampled households reported expenditures on alcohol averaging 7.3% of their total household budget. For the low income, the alcoholic drinks would tend to be cheaper and more hazardous types brewed at the household level or by illicit firms, obviating the possibility of using taxation as a policy option to regulate its consumption.

Evidence suggests that alcohol expenditures crowd out household expenditure on food and non-food commodities, with greater effects among poor households. Pu, Lan, Chou, and Lan [14] found alcohol expenditure to be significantly associated with lower expenditure on main food and other food for all income groups based on the expenditure data for Taiwan. Jumrani and Birthal [6] applying Engel’s law, confirmed the results using household data from India in which alcohol expenditure crowded out consumer durables more followed by food expenditure. They also found that the poor and socially disadvantaged households spent more on alcohol compared to other segments of society.

Previous literature on the crowding-out effect of temptation goods primarily focused on the effects of tobacco expenditure [6, 10, 11, 26]. Evidence on the role of alcohol expenditure has been limited owing to lack of data.

In the context of Malawi, even though reports, studies and other documentation have acknowledged the social and economic effects of alcohol consumption [1, 19, 20], the nature and extent of crowding out effects of alcohol consumption has not been documented. That said none of the available studies in Malawi have empirically analyzed alcohol consumption effects using econometric methods.

The present study quantifies the effects of alcohol consumption on household resource allocation in Malawi. The crowding out effects of alcohol consumption is evaluated using Quasi Maximum Likelihood Estimation (QMLE) methods. The method is appropriate for fractional response variables such as expenditure shares of different commodities that constitute total household consumption expenditure.

### 1.1 Methodological issues

#### 1.1.1 Intra-household resource allocation issues

In empirical work alcohol is consumed at the level of the individual, but analysis of its consumption demand is for practical purposes often done at the household level, which makes it necessary to clarify and justify the level of observation.

Traditionally, a consumer has been presented as a single individual but economic analysis using that unit has raised some queries for instance whether children can be consumers [27]. As a result, models that look at the consumer behavior have considered a consumer unit as comprising two or more people who make up a household. Those models are categorized into Unitary and Collective models [28]. Unitary models consider a household as a harmonious unit where a household is represented by a single person who makes decisions for the household and cares for everyone in the household. On the other hand, collective models divide the household into two actors with different interests which results in bargaining when making decisions [28].

However, considering a household as a consumer unit presents a limitation to a more detailed intra-household analysis of consumption effects but still due to unavailability of individual level consumption data, household level data has been widely used in literature when making such analyses. This study uses the household as a unit of analysis.

## 2 Analytical framework

The dependent variable in the models estimated in this study is a proportion or share of consumers’ spending on different categories of commodities. This restricts the variable into taking the values between 0 and 1. Having such kind of a dependent variable, a linear regression model generally yields nonsensical predictions for the extreme values of the regressors [29].

Appropriate models for analyzing such kind of a dependent variable have been suggested and replicated in several studies. The most widely used model to estimate the crowding out effects is the Quadratic Almost Ideal Demand System (QAIDS) [30]. The model estimates quadratic conditional Engel curves due to missing price information in the data. Considering that there is no truncation problem in the data as alcohol zero expenditure means that the data is censored at zero, the paper, hence, adopts a similar model that models zero values including all other values between 0 and 1. Papke and Wooldridge [31] developed this econometric method in which proportions data between 0 and 1 including intermediate values could be handled. In developing the functional forms, they assumed that, for all *i*,

$$E(y_{i}|x_{i})=G(x_{i}\beta )$$ 1

Where *G*(.) is a known function satisfying 0<*G*(*z*)<1 for all *z*∈ℝ. This ensures that the predicted values of *y* lie in the interval (0, 1). ***x***_***i***_: *i* = 1,2,…,*N* is a set of explanatory variables. To estimate Eq 1 they proposed a particular quasi-maximum likelihood estimator (QMLE). Given Bernoulli log-likelihood function as;

$$l_{i}(b)\equiv y_{i}\;\log\left[G(x_{i}b)\right]+(1-y_{i})log[1-G(x_{i}b)]$$ 2

QMLE of ***β*,** is obtained from the maximization problem

$${max}_{b}\sum_{i=1}^{N}l_{i}(b)$$

This Bernoulli QMLE $\hat{\beta }$ is consistent and asymptotically normal regardless of the distribution of *y*_*i*_ conditional on ***x***_***i***_; *y*_*i*_ could be a continuous variable, a discrete variable, or have both continuous and discrete characteristics. Recent developments in the generalized linear models (glm) estimation in Stata, has made it possible to handle models of fractional dependent variable. The glm approach makes use of the logit link function (that is, the logit transformation of the response variable) and the binomial distribution, which may be a good choice of family even if the response is continuous [29]. Synthesizing and expanding on the glm and quasi-likelihood, robust estimates and inference with fractional response variables are obtained [31].

Further, Wooldridge [32] extended the application to the fractional probit model with endogenous continuous explanatory variable. He set up endogeneity as an omitted variable problem assuming *y*_**2**_ is continuous endogenous variable:

$$E(y_{1}|z,y_{2},a_{1})=\Phi (x_{1}\beta_{1}+a_{1})$$ 3

$$y_{2}=z\delta_{2}+v_{2},$$

where *x*_**1**_ is a general nonlinear function of (***z***_**1**_, *y*_**2**_), *a*_**1**_ is an omitted factor thought to be correlated with *y*_**2**_ but independent of the exogenous variables ***z*.**

If (*a*_1_, *v*_2_) is jointly normal, Wooldridge [33] proposes a two-step control function method. In the first step *y*_**2**_ is regressed on ***z***_***i***_ and obtain the residuals, ${\hat{v}}_{i2}$. In the second “probit” of ***y***_***i*1**_ on ***x***_***i*1**_, ${\hat{v}}_{i2}$ are used to estimate parameters, a “generalized linear model” can also be implement instead.

With the possibility of endogeneity in alcohol expenditure and total household expenditure in excess of alcohol expenditure, the study follows Wooldridge’s [32] fractional probit model with endogenous continuous explanatory variable methodology. The empirical model thus is expressed as;

$$E(w_{i}|z,y_{i},a_{i})=\Phi (x_{1}\beta_{1}+a_{i})$$ 4

$$y_{i}=z\delta_{i}+v_{i},$$

Where:

$w_{i}=\frac{p_{i}q_{i}}{M}$ is the budget share of commodity *i* in the total household expenditure

*p*_*i*_, *q*_*i*_ and *M* are price for commodity *i*, quantity consumed for commodity *i*, and the total household expenditure excluding alcohol expenditure respectively

***z*** is a vector of exogenous variables including household characteristics

*y*_***i***_ captures endogenous expenditure on alcohol and total household expenditure in excess of alcohol expenditure

*a*_**1**_ is an omitted factor thought to be correlated with *y*_**2**_ but independent of the exogenous variables ***z*.**

### 2.1 Estimating crowding out effects

Deciding which estimation method and specification of the model should be used involves performing some tests which help make an informed decision on the best method and specification to use. In this study, these included a test for endogeneity of independent variables, a test for heteroskedasticity and a test for heterogeneity. Table 5 in the S1 Appendix reports the p-values obtained after performing these tests.

In the test for endogeneity, the Durbin Wu-Hausman test was used with the null hypothesis that the variables under suspicion of endogeneity are exogenous. The rejection of this null hypothesis implies that the variables are endogenous. The test was performed on each equation and the results show that only in the hotels equation are the variables exogenous. With endogeneity in alcohol expenditure and total household expenditure in excess of alcohol expenditure, the study follows Wooldridge’s [32] fractional probit model with endogenous continuous explanatory variable methodology.

Following the literature by [10, 11, 14, 34, 35], alcohol expenditure and total household expenditure in excess of alcohol expenditure are instrumented by adult ratio and total household expenditure respectively. As argued by Pu, Lan, Chou, and Lan [14] adult ratio has been used as an instrument on the basis that Malawi’s law forbids drinking under the age of 18, and no clear pattern of sex difference has been observed for alcohol expenditure.

Pagan-Hall test was used to test for homoscedasticity of errors in each equation with a null hypothesis that the errors are homoscedastic. Hence, rejecting the null hypothesis implies the existence of heteroskedasticity. The results indicate the presence of heteroskedasticity in the food and beverages, housing, furnishing, transport, communication, tobacco and hotels equations. With the presence of heteroskedasticity estimates are less efficient and standard errors are inconsistent, thus informing this study to employ an estimation method which deals with heteroskedasticity to produce more efficient estimates and consistent standard errors. The study, therefore, resolved to use bootstrapped standard errors with 1000 replications.

A test to examine whether preferences between alcohol consuming and alcohol non-consuming households are heterogenous was performed. The results inform the study on the specification of the model itself whether to allow for heterogeneity or not in the model. This test performed a joint significant test on the variables associated with the binary variable, *d*, which has values 1 for alcohol consuming households and 0 for non-alcohol consuming households. Hence, the null hypothesis is that *H*_0_: *σ*_1*i*_ = *σ*_2*i*_ = *σ*_3*i*_ = 0 in Eq 5. The rejection of the null hypothesis implies that the preferences are heterogenous and as such variables associated with *d* variables should be included in the model if one wants to allow for heterogeneity. The results indicate that the preferences are heterogenous in all equations except in food and beverages and education equations. Hence, the study allows for heterogeneity in the preferences between alcohol consuming and non-consuming households. The following is, hence, the final model that was estimated.

$$E(w_{i}|z,y_{i},a_{i},\theta_{i})=\Phi (x_{1}\beta_{1}+a_{i}+\theta_{i})$$ 5

$$y_{i}=z\delta_{i}+v_{i},$$

$$\theta_{i}=\sigma_{1i}d+\sigma_{2i}d(lnM)+\sigma_{3i}d(lnM{)}^{2}$$

where *θ*_*i*_ allows for unobserved heterogeneity

### 2.2 Alternative estimation technique

Generalized Method of Moments (GMM) Three Stage Least Squares (3SLS) GMM 3SLS has been used in literature to estimate crowding out effects of alcohol consumption when there exists endogeneity and heteroskedasticity. QMLE was preferred in this paper on the advantage that it was developed to specifically estimate models with dependent variable as a proportion. To ensure robustness of the QMLE results, GMM 3SLS results were obtained and compared. However, due to computational challenges as GMM 3SLS takes forever to converge, the GMM 3SLS results were obtained by running traditional 3SLS with 1000 bootstrap replications. Estimation results on the crowding out effects of alcohol consumption are reported in Table 4.

## 3 Data sources

Typical sources of data for this type of study would be household surveys. For less developed countries Integrated Household Surveys have become a standardized progress monitoring instrument. Malawi’s National Statistical Office (NSO) conducts national household surveys every five years mainly to provide benchmark poverty, vulnerability, and socio-economic indicators to foster evidence-based policy formulation and monitor the progress of meeting various development goals. The present study uses data from the Fourth Integrated Household Survey (IHS4) which used a stratified two-stage sample design where primary sampling units (PSU) sampled in the first stage were Enumeration Areas (EAs) and households from each EA were sampled in the second stage. IHS4 covered 779 EAs each with an average of about 235 households. A total of 12480 households were sampled.

## 4 Preliminary results: Expenditure orientation

A dummy variable taking the values of 1 and 0 divides the sample into alcohol consumers (1) and non-consumers (0). Table 1 reports the summary averages for total household consumption expenditure, per capita consumption expenditure, alcohol expenditure and alcohol budget share for consumers and non-consumers in the full sample, the urban and rural samples. The sample was further divided into different expenditure groups by percentiles. The 1^st^ to 29^th^ percentile of the distribution of total household consumption expenditure represents the lower-income group, while those between 30^th^ and 70^th^ percentile are in the middle-income group and those above the 70^th^ percentile represent the higher-income group.

**Table 1:** Summary averages.

	Full sample		Rural		Urban	
	Consumers	Non-consumers	Consumers	Non-consumers	Consumers	Non-consumers
**All income**						
Average household expenditure	1180665	787094.3	997350.1	619566.9	1667916	1571871
Average per capita expenditure	380024	209033.7	319551.7	160329.2	540760	437188.8
Average alcohol expenditure	64283.83	0	56977.76	0	83703.41	0
Average alcohol budget share (%)	7.51	0.00	7.97	0.00	6.30	0.00
**Low income**						
Average household expenditure	310785.9	296313.9	307054.7	295766.2	343504.9	308398.3
Average per capita expenditure	174760.8	119335.4	165987.9	116811.1	251692.8	175035.8
Average alcohol expenditure	26861.94	0	26768.43	0	27681.95	0
Average alcohol budget share (%)	9.06	0.00	9.13	0.00	8.47	0.00
**Middle income**						
Average household expenditure	621425	587980.4	619514.7	583133.2	634075.1	626421.3
Average per capita expenditure	175985.1	159015.5	157951.7	150046.4	295405.9	230145.4
Average alcohol expenditure	49283.72	0	49939	0	44944.31	0
Average alcohol budget share (%)	7.98	0.00	8.08	0.00	7.33	0.00
**High income**						
Average household expenditure	1986782	1578154	1944324	1235406	2036357	2083816
Average per capita expenditure	636167.4	372923.7	644616.3	254468.7	626302.3	547681.9
Average alcohol expenditure	90677.47	0	84410.51	0	97994.96	0
Average alcohol budget share (%)	6.56	0.00	7.16	0.00	5.86	0.00

Notes: Expenditures are measured in Malawi kwacha (MWK), a local currency in Malawi, as of January 21, 2022, it is valued at 816.4 Malawi Kwacha to 1 USD

Overall, alcohol-consuming households, have higher total household expenditure and per capita expenditure in all sub-samples. In addition, within alcohol-consuming households, as one moves from lower-income category to higher-income category both total household expenditure and per capita expenditure increase in all sub-samples as well. Furthermore, alcohol budget share is proportionately higher for rural households than urban households; rural households, on average spent as much as 7.97% of their total budget on alcohol while urban households, on average spent about 6.3% of their household budget on alcohol. Additionally, alcohol expenditure budget share is proportionately higher among the poor than their richer counterparts. Average alcohol budget share declines as one moves from lower-income category to a higher-income category in all sub-samples. This is an indication that if alcohol is to burden the consuming households, poor households are at a higher risk than their richer counterparts.

Table 2 presents Student’s *t*-test for the differences in mean expenditure shares between alcohol-consuming and non-consuming households for the full sample. Testing for the mean differences on expenditure shares gives a preliminary indication on potential crowding out effects. Positive mean differences imply that alcohol non-consuming households spend more on that consumption good than alcohol consuming households, while negative mean differences imply the opposite. Student’s *t-*test is a two-sample test for the equality of mean with a null hypothesis that the difference in mean expenditure share = 0. This study uses the weighted mean expenditure shares computed using the survey weight provided in IHS4.

**Table 2:** T-Test for the differences in mean of shares between alcohol consumers and non-consumers for the full sample.

Commodity categories	Consuming households	Non-consuming households	Difference in mean expenditure share
Food & Beverages	0.5681	0.6147	0.0465 ***
Clothing & footwear	0.0247	0.0209	-0.0038**
Housing	0.1557	0.1948	0.0390***
Furnishing	0.0326	0.0356	0.0029**
Health	0.0209	0.0185	-0.0023
Transport	0.0398	0.0343	-0.0055
Communication	0.0208	0.0213	0.0004
Recreation	0.0042	0.0029	-0.0013**
Education	0.0128	0.0170	0.0042**
Hotels	0.0153	0.0100	-0.0053***
Others	0.0254	0.0295	0.0040***
Tobacco	0.0036	0.0006	-0.0030***
Durables	0.0409	0.0420	0.0011

Notes: Food and Beverages category does not include alcohol. When constructing consumption aggregates alcohol was separated from beverages category

***, **, * represents 1%, 5% and 10% significance level

For the full sample alcohol non-consuming households significantly and proportionately spent more of their budget than the consuming households as follows: on food and beverages, 61.47% vs 56.81%; on housing 19.48% vs 15.57%; on furnishing 3.56% vs 3.26%, on education 1.7% vs 1.28%. and on “other” expenditures 2.95% vs 2.54%. The differences are significant at the 1% level of significance except furnishing and education which are significant at the 5% level. Alcohol consuming households significantly and proportionately had more of their budgets going to vanity-oriented expenditures than none consuming households as follows: on clothing 2.47% vs 2.09%; on recreation 0.42% vs 0.29%, on hotels 1.53% vs 1%, and; on tobacco 0.36% vs 0.06%. Worth noting is that mean difference in expenditures on durables is insignificant in the full sample implying that consuming and non-consuming households do not significantly spend differently on durable goods. Other consumption goods with insignificant mean expenditure differences are heath, transport and communication.

Table 3 which reports results for the Student’s *t-*test for the rural and urban sample, shows that the consuming and non-consuming households in the rural area significantly and proportionately spend differently on food and beverages, housing, furnishing, health, communication, education, hotels, others, tobacco and durables. Their mean expenditure differences in clothing and footwear, transport and recreation are not significantly different. Whilst the urban sample, significantly (at the 1% level) and proportionately spend differently only on food and beverages, housing, hotels, others and tobacco in favour of non-consuming household.

**Table 3:** T-Test for the differences in mean of shares between alcohol consumers and non-consumers for Rural and Urban households.

Commodity categories	Consuming households		Non-consuming Households		Difference in mean expenditure share	
	Rural	Urban	Rural	Urban	Rural	Urban
Food & Beverages	0.6006	0.4905	0.6332	0.5319	0.0326***	0.0414***
Clothing & footwear	0.0208	0.0340	0.0189	0.0298	-0.0019	-0.0041
Housing	0.1488	0.1723	0.1920	0.2032	0.0431***	0.0349***
Furnishing	0.0314	0.0356	0.0356	0.0365	0.0040***	0.0008
Health	0.0236	0.0145	0.0192	0.0159	-0.0045*	0.0014
Transport	0.0273	0.0699	0.0296	0.0557	0.0023	-0.0141
Communication	0.0147	0.0355	0.0176	0.0377	0.0029**	0.0022
Recreation	0.0016	0.0105	0.0016	0.0088	0.0000	-0.0017
Education	0.0087	0.0225	0.0143	0.0287	0.0057***	0.0062
Hotels	0.0138	0.0187	0.0093	0.0130	-0.0046***	-0.0057**
Others	0.0242	0.0284	0.0284	0.0343	0.0042***	0.0059***
Tobacco	0.0038	0.0032	0.0006	0.0004	-0.0032***	-0.0028***
Durables	0.0249	0.0523	0.0367	0.0526	0.0118**	0.0003

***, **, * represents 1%, 5% and 10% significance level

## 5 Estimation results; Crowding out effects

Table 4 reports the results of the effect of alcohol expenditure on the expenditure of all other commodities. Significant negative coefficients provide evidence of crowding out effects of alcohol consumption expenditure on household’s resource allocation. The coefficients were estimated using Quasi Maximum Likelihood Estimation (QMLE) in the Generalized Linear Model (GLM) framework.

**Table 4:** QMLE estimation results for the crowding out effects of alcohol.

	Full Sample			Urban			Rural		
	OLS	GMM 3SLSS	QMLE	OLS	GMM 3SLSS	QMLE	OLS	GMM 3SLSS	QMLE
Food & Beverages	-0.000014 *** (0.0000028)	-0.000018** (0.00000092)	-0.000065*** (0.00000648)	-0.000020** (0.0000063)	-0.000025 (0.000015)	-0.000029** (0.0000115)	-0.000011*** (0.0000031)	-0.000011** (0.0000046)	-0.000057*** (0.0000071)
Clothing & footwear	0.0000049*** (0.0000007)	0.0000045** (0.0000023)	-0.000020** (0.00000817)	0.00000069*** (0.0000017)	-0.0000049 (0.0000030)	-0.000021 (0.0000144)	0.0000044*** (0.00000084)	0.0000031** (0.0000013)	0.0000091 (0.0000133)
Housing	0.0000034** (0.0000016)	-0.0000049* (0.0000029)	0.000036*** (0.00000453)	0.0000011 (0.0000043)	0.0000037 (0.0000032)	-0.000036*** (0.0000092)	0.0000026* (0.0000014)	-0.0000027** (0.0000013)	0.000020*** (0.0000048)
Furnishing	-0.0000006 (0.0000007)	-0.0000012 (0.00000083)	0.000011 (0.0000102)	0.0000033 (0.0000022)	-0.0000011 (0.0000013)	0.0000023 (0.0000277)	-0.0000014* (0.00000074)	0.0000013** (0.00000072)	0.0000069 (0.000010)
Health	0.0000005 (0.0000011)	0.0000056** (0.0000028)	0 .000022* (0.0000115)	0.00000037** (0.0000017)	-0.0000032 (0.0000022)	-0.000011 (0.0000221)	0.0000049*** (0.0000012)	0.0000045** (0.0000019)	0.000032** (0.0000161)
Transport	0.0000034** (0.0000016)	0.0000036* (0.0000022)	0.0000088 (0.0000116)	0.0000079* (0.0000041)	-0.0000041 (0.0000031)	0.000050** (0.0000209)	0.0000022 (0.0000017)	0.0000021* (0.0000013)	-0.0000198 (0.0000167)
Communication	-0.000002** (0.00000076)	-0.0000026* (0.0000014)	-0.00014*** (0.0000096)	-0.0000046** (0.0000019)	-0.0000051 (0.0000035)	-0.000132*** (0.0000147)	-0.0000014 (0.00000084)	-0.0000014* (0.00000078)	-0.000108*** (0.0000122)
Recreation	-0.00000045* (0.00000024)	-0.00000058* (0.00000034)	-0.000088*** (0 .0000131)	-0.00000084 (0.00000097)	0.00000097 (0.00000067)	-0.000113*** (0.0000154)	0.00000034* (0.00000021)	-0.00000033* (0.00000018)	-0.000060** (0.0000235)
Education	-0.000021*** (0.00000089)	-0.000022** (0.000011)	-0.00017*** (0.0000118)	0.000033*** (0.0000034)	0.000026* (0.000016)	-0.000200*** (0.0000252)	-0.000018*** (0.0000008)	-0.000015** (0.0000058)	-0.000239*** (0.0000186)
Hotels	0.00000013** (0.00000045)	0.00000074 (0.00000051)	-0.000037** (0.0000114)	0.00000003 (0.00000011)	0.00000038 (0.00000050)	0.0000014 (0.0000242)	0.0000017** (0.0000005)	0.00000076* (0.00000041)	-0.000023* (0.0000123)
Others	-0.0000002*** (0.00000044)	-	-0.000036*** (0.0000043)	-0.0000025** (0.000001)	-	-0.000040*** (0.0000100)	- 0.0000016** (0.00000049)	-	-0.000041*** (0.0000054)
Tobacco	0.0000007** (0.00000024)	0.00000014 (0.00000013)	0 .0000216 (0.0000254)	0.00000028 (0.00000061)	0.00000009 (0.00000019)	0.0000014 (.0000879)	0.00000072** (0.00000028)	0.00000014 (0.000000095)	0 .000059* (0.0000326)

Notes: The results in this table are coefficients of alcohol real expenditure variable. The estimated values were controlled for household size, location, gender, characteristics of household head (age, educational attainment, and religion). Observations: Full sample: 12,447 households, Urban: 2,272, Rural: 10.175; Bootstrapped standard errors in parathesis for QMLE and heteroscedasticity-robust standard errors for OLS; “Others” consumption category was left out for GMM 3SLS as commonly done in the literature (John, et al., 2019)

***, **, * represents 1%, 5% and 10% significance level respectively.

Alcohol consumption expenditure is measured as total household real expenditure on alcohol in local currency, Malawi kwacha (MWK). Expenditure shares of all other commodities are also measured in Malawi kwacha, constructed as total consumption on each commodity category divided by the total household consumption expenditure in excess of alcohol expenditure.

To obtain robust results, firstly we begin with the linear version of Eq 5 which is estimated by ordinary least squares (OLS) for the full, urban, and rural samples. Secondly, Eq 5 is also estimated by traditional 3SLS with 1000 bootstrap replications to similar results to GMM 3SLS. Finally, using probit QMLE, Eq 5 is estimated as a non-linear model for the full, urban, and rural samples as well. All the results are presented in Table 4.

Since this study is interested in estimating the crowding out effects of alcohol consumption expenditure, the study does not devote much attention on reporting and interpreting the coefficients of household characteristics variables. Household characteristics variables which were included in the estimation model include age, education, marital status, gender and religion of the household head, household size and location (rural or urban).

OLS results show that alcohol expenditure crowds out household expenditure on food and beverages, education and “others” in all samples. Communication expenditure was significantly crowded out by alcohol consumption expenditure in the urban sample. On the other hand, no significant crowding out effects were observed in urban sample from GMM 3SLS results. However, just like in the OLS estimation results, food and beverages expenditure was crowded out by alcohol consumption expenditure in all samples. Additionally, GMM 3SLS results show that alcohol consumption expenditure crowds out household expenditure on housing, communication, recreation and education in the full and rural samples.

The main results from QMLE in the full sample show that on average, an increase in alcohol consumption expenditure leads to a decrease in budget share devoted to food and beverages, clothing and footwear, communication, recreation, hotels, education and “others”. Alcohol consumption expenditure consistently crowds out expenditure on food and beverages from OLS, GMM 3SLS and QMLE results, while communication, recreation and education expenditures were consistently crowded out in the full and rural samples from GMM 3SLS and QMLE results. In the urban sample the other important expenditure that was crowded out is the expenditure on housing. These results are similar to those found by Pu, Lan, Chou, and Lan [14] in which alcohol expenditure was significantly associated with lower expenditure on main food, other food, utilities, housework and education.

Education expenditure is the most crowded out expenditure in all samples as shown in both OLS and QMLE results. The results corroborate the findings of Jumrani and Chaloupka [6] who also found that alcohol consumption expenditure crowds out education among other consumption goods they considered. This result has significant implication on the economy especially on the infant economy like that of Malawi in terms of human capita development and economic growth. Education is considered as a long-term investment that leads to rise in productivity in future. Spending on education helps to promote efficiency, knowledge, and inventions, all of which contribute to the economic growth and development of a nation [36]. Hence, this result is a revelation about the distortions of alcohol consumption expenditure through crowding out education expenditure on Malawi’s economy.

Another important expenditure in relation to alcohol consumption expenditure is health expenditure. A somewhat expected result has been found that increase in alcohol consumption expenditure on average increases the share of the expenditure on health on total household expenditure. Alcohol consumers are exposed to health risks associated with chronic heavy drinking such as injuries and chronic diseases. As such, their expenditure on health is expected to increase with increasing expenditure on alcohol consumption.

## 6 Conclusion

It has been found that alcohol consumption expenditure is an important expenditure and crowds out other necessary expenditures. The findings indicate that the consumption of alcohol is at the expense of consumer non-durable (food and beverages), durable (housing) and essential services (education) expenditures. This implies negative impacts on the wellbeing or even an escalation of poverty through misallocation of household resources–however a trend which merits monitoring. This goes against poverty eradication by 2030, one the five priority areas of the Malawi Growth and Development and Strategy (MGDS) III and which are aligned to the Sustainable Development Goals (SDGs).

Due to the estimated crowding out effects of alcohol consumption expenditure, reduced consumption of alcohol, whether by quitting, reduced frequency of use, may have positive implications on households’ resource allocation on other goods, such as food, housing, education and overall quality of life. This implies a definite need for policy attention towards alcohol consumption expenditure. Price mechanisms for reducing alcohol consumption such as taxation could be used to regulate or reduce alcohol consumption.

A key limitation of the study is that it is based on the “household” as the unit of analysis, whereas much of the alcohol consumption decisions are made at the individual level requiring intra-household analysis of alcohol consumption expenditure. This lack of intra-household resource allocation information is a blind spot of the now generic household welfare surveys. Additionally, the data captured in the Integrated Household Survey (IHS) is based on self-reported information for a previous recall period, creating room for measurement error. Furthermore, direct price information is not provided in IHS surveys.

## Supporting information

S1 Appendix (DOCX)
