# Supplementary material for: Crowding Out Effects of Alcohol Consumption Expenditure on Household Resource Allocation in Malawi
Source: PLoS One. 2022 Feb 4;17(2):e0263330. doi: 10.1371/journal.pone.0263330 (PMC8815938; doi:10.1371/journal.pone.0263330)
Supplement: S1 Appendix — (DOCX) [file pone.0263330.s001.docx]

## **Appendix**

**Table 5. Test results from diagnostic tests**

|  | **P-values** | | |
| --- | --- | --- | --- |
| **Commodity category** | **Endogeneity test**  (Durbin Wu-Hausman test) | **Heteroskedasticity test**  (Pagan-Hall test) | **Heterogeneity test**  (Joint significance of d, d(lnM) & d${(lnM)}^{2}$ |
| Food & Beverages | 0.0487**  0.0490** | 0.0000*** | 0.8420 |
| Clothing & footwear | 0.0000*** | 0.9361 | 0.0092** |
| Housing | 0.0009** | 0.0000*** | 0.0018** |
| Furnishing | 0.0000*** | 0.0109** | 0.0002*** |
| Health | 0.0000*** | 0.6132 | 0.1584 |
| Transport | 0.0024** | 0.0000*** | 0.0013** |
| Communication | 0.0690*  0.0694* | 0.0000*** | 0.0995* |
| Recreation | 0.0000*** | 0.0546 | 0.0002** |
| Tobacco | 0.0000*** | 0.0000*** | 0.0000*** |
| Education | 0.0000*** | 0.7624 | 0.4709 |
| Hotels | 0.2665 | 0.0000*** | 0.0268** |
| **Null hypothesis** | Variables are exogenous | Disturbance is homoscedastic | Preferences are homogenous |
| **Alternative hypothesis** | Variables are endogenous | Disturbance is heteroskedastic | Preferences are heterogenous |
| ***, **, * indicate the p-value of less than 1%, 5% and 10% respectively | | | |
